# Key role of exopolysaccharide on di-butyl phthalate adsorbing by *Lactobacillus plantarum* CGMCC18980

**DOI:** 10.1007/s00253-021-11145-w

**Published:** 2021-03-05

**Authors:** Yu-Hang Fan, Yi-Lin Shen, Zhi-Wei Lin, Ying Zhou, Bang-Ce Ye

**Affiliations:** 1 Department of Food Science and Technology, School of Bioengineering, East China University of Science and Technology, Meilong RD 130, Shanghai, 200237 China; 2 Lab of Biosystems and Microanalysis, State Key Laboratory of Bioreactor Engineering, East China University of Science and Technology, Meilong RD 130, Shanghai, 200237 China

**Keywords:** *Lactobacillus plantarum*, Di-butyl phthalate, Adsorption, Estrogen

## Abstract

**Abstract:**

Plasticizers belong to hormone-like substances existing widely in the environment. According to the Environmental Protection Agency of China, they are considered to be the fourth class of toxic chemicals due to their harmful effects on normal endocrine system in human bodies. In the recent published work of our lab, *Lactobacillus plantarum* CGMCC18980 (strain P1) could reduce the toxicity of di-butyl phthalate (DBP) in rats effectively. The purpose of this study is to further explore the adsorption mechanism of di-butyl phthalate to *L. plantarum* CGMCC18980, based on optimizing the adsorption conditions. As a consequence, the adsorption effect of *L. plantarum* CGMCC18980 attributed to relationships between exopolysaccharide, membrane protein, and the cell wall. Experimental results demonstrated that exopolysaccharide and the cell wall were devoted to DBP binding. An obvious adsorption layer was observed outside of *L. plantarum* CGMCC18980 through scanning electron microscope (SEM) and transmission electron microscope (TEM). The Fourier transform infrared spectroscopy (FTIR) results showed that the functional groups involved in adsorption were mainly C=O, C-N, and C-O, which related to lipids and polysaccharides. Zeta potential analysis indicated that DBP adsorption had no significant relationship with surface charge. These results revealed that exopolysaccharide may be the key factor of strain CGMCC18980 in DBP adsorption.

**Key points:**

• *Lactobacillus plantarum CGMCC18980 has the ability to adsorb di-butyl phthalate, reaching to 58.63%.*

• *Exopolysaccharide is considered to play a key role in adsorption process.*

• *Membrane protein, cell wall, and surface charge do not contribute to adsorption.*

## Introduction

Phthalates (PAEs), as the most commonly used plasticizers in the market at present, can reach 20–50% of the product (Gao et al. 2014). With the migration of time, such substances are easy to accumulate through the food chain and difficult to degrade in the natural environment (Wang and Chen 2009). Moreover, direct exposure to a phthalate mixture adversely can affect antral follicle health in vitro (Zhou and Flaws 2017). Di-butyl phthalate (DBP), one of the plasticizers, is most commonly used in polyvinyl chloride (PVC) processing, which can lead to reproductive tract malformation in male rats during sexual differentiation (Howdeshell et al. 2007), affecting the secretion of human sex hormones and threatening human health (Ghisari and Bonefeld-Jorgensen 2009; Swan 2008). Meanwhile, DBP was proved to be harmful to rodents (Higuchi et al. 2003), and further studies showed that DBP could induce antiandrogenic effects by inhibiting steroidogenic factor 1 (SF1) indirectly (Plummer et al. 2013). Some research demonstrated that the exposure to DBP disrupted ovarian function in animal models and in human cells in vitro (Adir et al. 2017). DBP is such a kind of ubiquitous harmful substance that the correct treatment of it is particularly crucial. Traditional methods to dispose those substances cannot completely remove DBP so that simple and efficient methods are sought to solve this problem urgently.

Biosorption is a hot topic in recent years, and some microorganisms generally regarded as safe are commonly used in this respect, involving lactic acid bacteria (LAB). Some strains of LAB have shown the ability in removing harmful and reducing toxicity, mainly concentrated on harmful heavy metal ions and some fungal toxins (Ge et al. 2017; Shen et al. 2018; Wang and Chen 2009; Baralić et al. 2020; Al-Enazi et al. 2020). There are some researches on the adsorption of DBP by LAB (Zhao et al. 2017), but its mechanism is still lacking.

In this paper, DBP, a highly toxic phthalate ester, was chosen as the study object. The preliminary results in our laboratory showed that *L. plantarum* CGMCC18980 could reduce the concentration of DBP in rats (Shi et al. 2020). This study tried to find out the adsorption mechanism of *L. plantarum* CGMCC18980 based on bacterial cells composition, FTIR analysis, and direct electron microscope observation. At the same time, this research may fill in the gaps in this field and provide new ideas which have great potential and may bring changes to the food industry in the future. Besides, *L. plantarum* CGMCC18980 may be used as a DBP biosorbent or a potential drug to ameliorate its toxicity, and has broad application prospects, especially in the current complex and changeable circumstances.

## Material and methods

### Strains and cultivation conditions

*L. plantarum* CGMCC18980 was screened from Xinjiang dairy products in our laboratory (Hu et al. 2019). *L. plantarum* LP-115 was selected as control, presented DuPont Danisco (Shanghai, China). All strains were stored in glycerin at −18°C at 25% (v/v). Two strains were cultured in MRS broth (Solarbio Co., Beijing, China). DBP (200 mg/L, dissolved in methanol) was purchased from Huawei Ruike (Beijing, China), and liquid-phase methanol from Titan (Shanghai, China). The reagents used in this study were all of analytical-reagent grade.

### Preparation of bacterial cells

After activation, 1.00% *L. plantarum* were cultured in MRS liquid medium and inoculated at 30 °C, 200 rpm for 24 h. Both two strains were cultured in MRS medium at 30 °C for 24 h under anaerobic condition (Hernandez-Mendoza et al. 2009). The obtained bacterial cells could be used for subsequent experimental operations.

### DBP-binding assay

The bacteria cells were harvested by centrifugation (4000 rpm, 10 min), washed with 0.90% saline at least three times, and finally adjusted to OD_600_=7.00. DBP and bacterial cells were added into the test tube and then shaken evenly. The samples were incubated without shaking. The adsorption ratio was determined under different conditions. The adsorption system was centrifuged after the incubation (4000 rpm, 10 min), and the upper layer was transferred to another tube. Ethyl acetate of the same volume was used for extraction and the supernatant was dried. Finally, the residue was dissolved in 1.00 mL methanol (Zoghi et al. 2019). The residual amount of DBP was determined by high-performance liquid chromatography (HPLC), with the equivalent amount of DBP (200 mg/L) taken as the control.

### HPLC conditions

The concentration of DBP was measured on an HPLC system (Shimadzu Nexera LC). The C18 column (250×4.6 mm I.D., 5 μm; Teknokroma) was equilibrated with methanol/water (90:10, v/v) as the mobile phase. Ten microliters of each sample was injected and eluted with a flow rate of 1.00 mL per minute at 40 °C, and UV detection was done at 254 nm (Zhu et al. 2013; Wang and Chen 2009). The adsorption ratio of bound DBP was calculated with following equation:$Y=\left(1-\frac{A}{A_0}\right)\ast 100$, where *Y* is the adsorption ratio of DBP, *A* is the peak area of DBP in the supernatant, and *A*_0_ is the peak area of DBP in blank sample.

### Effect of exopolysaccharide and membrane protein on DBP adsorption

The bacteria cells (25.00 mL) were centrifuged (4000 rpm, 10 min) and recovered in 1 mol/L NaCl solution. Ultrasonication (78 W, 3 min 10 °C) was applied to separate exopolysaccharide from cells (Hernandez-Mendoza et al. 2009), and the bacteria cells were obtained after centrifugation (4000 rpm, 5 min, 4 °C), and then measured the adsorption ratios. The membrane protein can also be removed. Dissolve the cells in lithium chloride solution (25.00 mL, 5 mol/L) at 4 °C for 60 min after centrifugation (Smit et al. 2001). The bacteria cells without membrane protein can be obtained after centrifugation (4000 rpm, 5 min, 4 °C). Determine adsorption ratios according to the previous method and utilize the original bacteria cells as the control.

### Effect of cell wall on DBP adsorption

The bacterial cells were centrifuged (4000 rpm, 5 min, 4 °C), and the mixtures were prepared through ultrasound at 30 °C for 30 min (power 400 W, working for 3 s, intermittent 7 s), followed with the centrifugation (10,000 rpm, 10 min, 4 °C) to get the crude cell wall extract (Yamamoto et al. 2000). The crude cell wall extract was added to 10 mL 8% SDS and placed in boiling water for 10 min. After that, it was quickly cooled to room temperature and centrifuged (10000 rpm, 20 min, 4 °C). The precipitate was collected and washed twice with sterile deionized water, and then dissolved in 0.10 mol/L Tris-HCl buffer, adjusting pH to 7.60. Add 3 mg/mL trypsin and incubate at 37 °C overnight. The cell wall extract was obtained after centrifuging (10000 rpm, 20 min, 4 °C) and washed twice in saline. Determine the DBP adsorption ratios as mentioned above and original bacteria cells as the control.

### FTIR analysis and zeta potential analysis of bacterial cells

To determine the potential functional groups and putative binding sites related to DBP adsorption, FTIR 6700 (Thermo Nicolet Corporation) was carried out to the analysis. After being dried in a freeze dryer FD-2 from Bilon (Shanghai, China) for 48 h, the bacterial cells sample (dry weight) and KBr powder were mixed and grind in an agate mortar (KBr: sample =100:1), and then 30.00 mg of each sample mixture was pressed into a transparent plate (Dan et al. 2010; Lin et al. 2011). All infrared spectra ranging from 4000 to 400 cm^−1^ were recorded at room temperature. The changes of surface charge in cells before and after adsorption can be analyzed by zeta potential. The bacterial cells were diluted in 10 mL tube after incubation and centrifugation. Then use the micro electrophoresis apparatus Zeta Plus (Zetasizer 3600; Malvern Instruments, UK) to measure at room temperature (25 °C, pH 4.00). All samples were determined under the same experimental conditions (*n*=30) (Jastrzębska et al. 2015).

### Characteristics of bacterial cells

#### Scanning electron microscope (SEM) analysis

Scanning electron microscope S-3400N (Hitachi, Japan) was used for observation and photography. Bacterial cells were dried overnight in a drying dish (Bergmans et al. 2005) through gradient dehydration with ethanol (30–100%). All the cell samples were observed under 15 kV.

#### Transmission electron microscope (TEM) analysis

Biological transmission electron microscope JEM-1400 (Hitachi, Japan) was applied for observation and imaging. The bacterial cells were diluted to a certain ratio and dropped into the copper mesh, and then they were observed under TEM (Huang et al. 2011). The thickness of exopolysaccharide in the cell wall was measured by digital micrograph software (Gatan, American).

### Statistical analysis

All experimental samples were carried out in triplicate, and the data were expressed as mean ± standard deviation (SD), while significant differences were analyzed with *t* test. GraphPad Prism 7.0 was used to generate graphs and conduct data analysis.

## Results

### DBP-binding assay

**Fig. 1:**
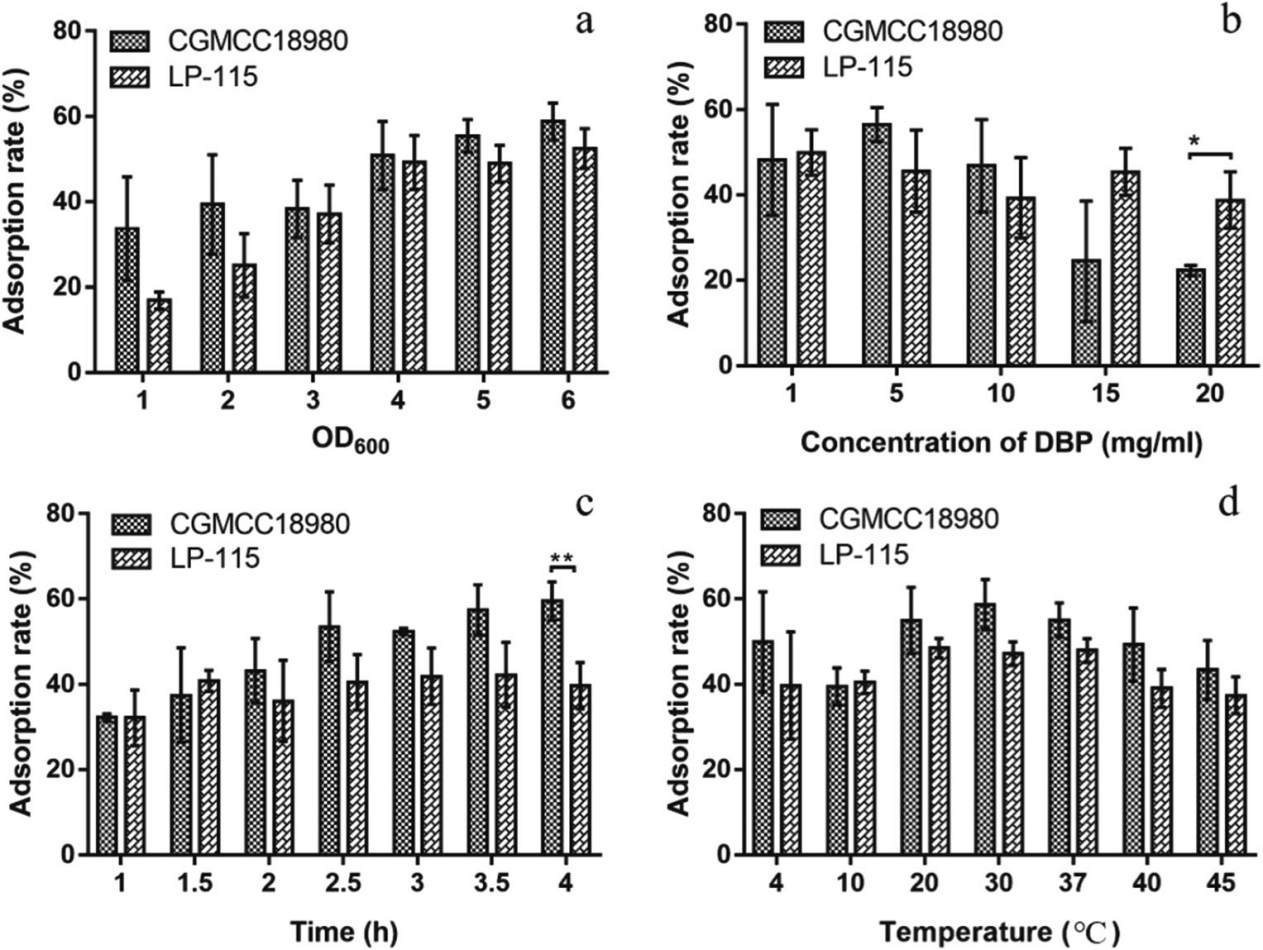
Adsorption ratios of DBP in different conditions (“*” means *P*<0.05): **a** OD_600_. **b** Concentration of DBP (mg/mL). **c** Time (h). **d** Temperature (°C)

**Fig. 2:**
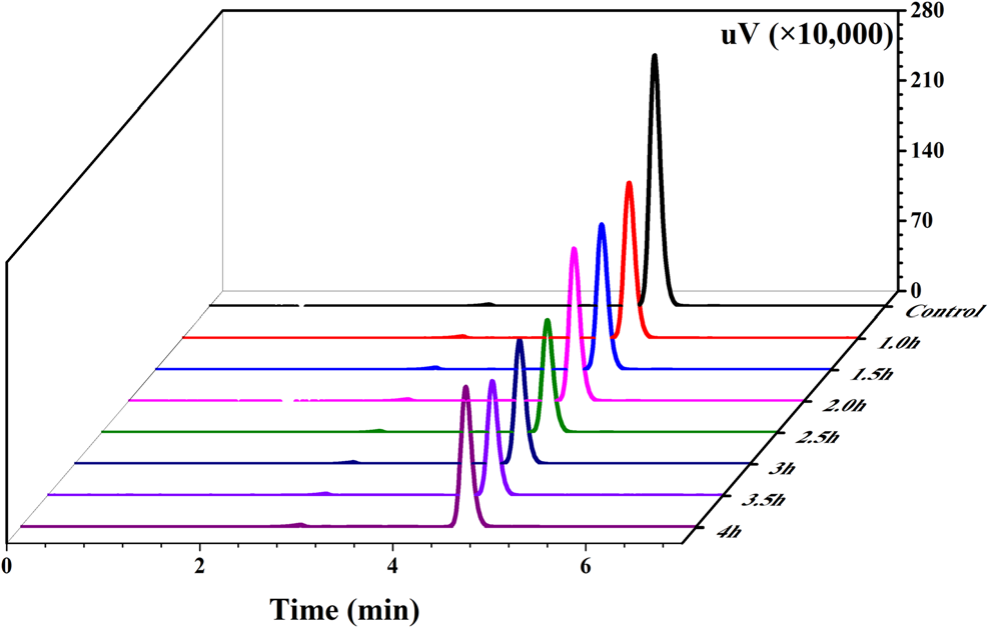
Liquid chromatogram of DBP at 30 °C in different times (use no bacteria group as control). The remain time is about 4.5 min at 254 nm, regard as DBP

The DBP-binding ratios of two strains were displayed in Fig. 1. CGMCC18980 showed higher adsorption ratios at a lower bacterial concentration (OD_600_ < 2.00) in this experiment (Fig. 1a). In higher cell concentration, the condition was similar, and the ratios of CGMCC18980 and LP-115 were determined to be 50.18% and 49.22% at OD_600_=4.00, respectively. The ratios increased at the low bacterial concentration (OD_600_≤4.00), while with the increasing of cells, this trend changed slightly (Fig. 1a). OD_600_ of 4.00 was selected for subsequent experiments based on the results. DBP concentrations also had a great influence on the adsorption ratio. The concentration of 5.00 mg/mL had the highest adsorption ratio of 56.53% and 45.57% in CGMCC18980 and LP-115 (Fig. 1b), respectively. When DBP concentration reached 20.00 mg/mL, there was a significant difference between CGMCC18980 and LP-115 (*P*<0.05) with a sharp drop in the adsorption ratio of CGMCC18980 (Fig. 1b). The metabolites of DBP in different time were also detected in this study, but there were no metabolites during the incubation with CGMCC18980 (Fig. 2). It indicated that *L. plantarum* CGMCC18980 did not degrade DBP but only adsorbed DBP.

In order to explore the reaction speed, the adsorption ratios under different time and temperature were measured (Fig. 1c and d). After 2.50 h exposure to DBP, the ratio of *L. plantarum* CGMCC18980 attained 53.46%, while LP-115 was 40.45% (Fig. 1c). In the early stage of the experiment, the adsorption ratio of CGMCC18980 increased steadily, but the adsorption ratio did not change significantly after 2.50 h (Fig. 1c). As shown in Fig. 2, during the 4 h adsorption incubation process, the peak area indicated the residual DBP content. The peak area decreased from 2282115 in the control group to 11688576 after 4 h, indicating that the concentration of DBP in the system decreased from 200 to about 104.90 mg/L. Moreover, after 2.5 h, the peak area in the chromatogram did not change significantly. This meant that most of DBP was adsorbed after the 2.50 h in the experiment of 4 h. Besides, the ratios could be affected by temperature. In the range of 4 to 45 °C, the adsorbing ratio of CGMCC18980 was 39.48 to 58.63%, and that of LP-115 was 37.46 to 48.44%. The CGMCC18980 had the highest adsorption ratio of 58.63% at 30 °C, followed by 54.91% at 37 °C, while LP-115 showed the lower ratios of 47.14% and 48.44% at 37 °C and 30 °C, respectively (Fig. 1d). Thirty degrees Celsius was selected in subsequent experiments. The above results suggested that CGMCC18980 was more capable than LP-115 in adsorption of DBP.

According to the previous experimental results, two strains were selected to adsorb DBP with the condition of OD_600_=4.00, 5.00 mg/mL concentration of DBP, 2.50 h and 37 °C as the optimum conditions in subsequent experiments.

### Effect of exopolysaccharide, membrane protein, and cell wall on DBP adsorption

**Fig. 3:**
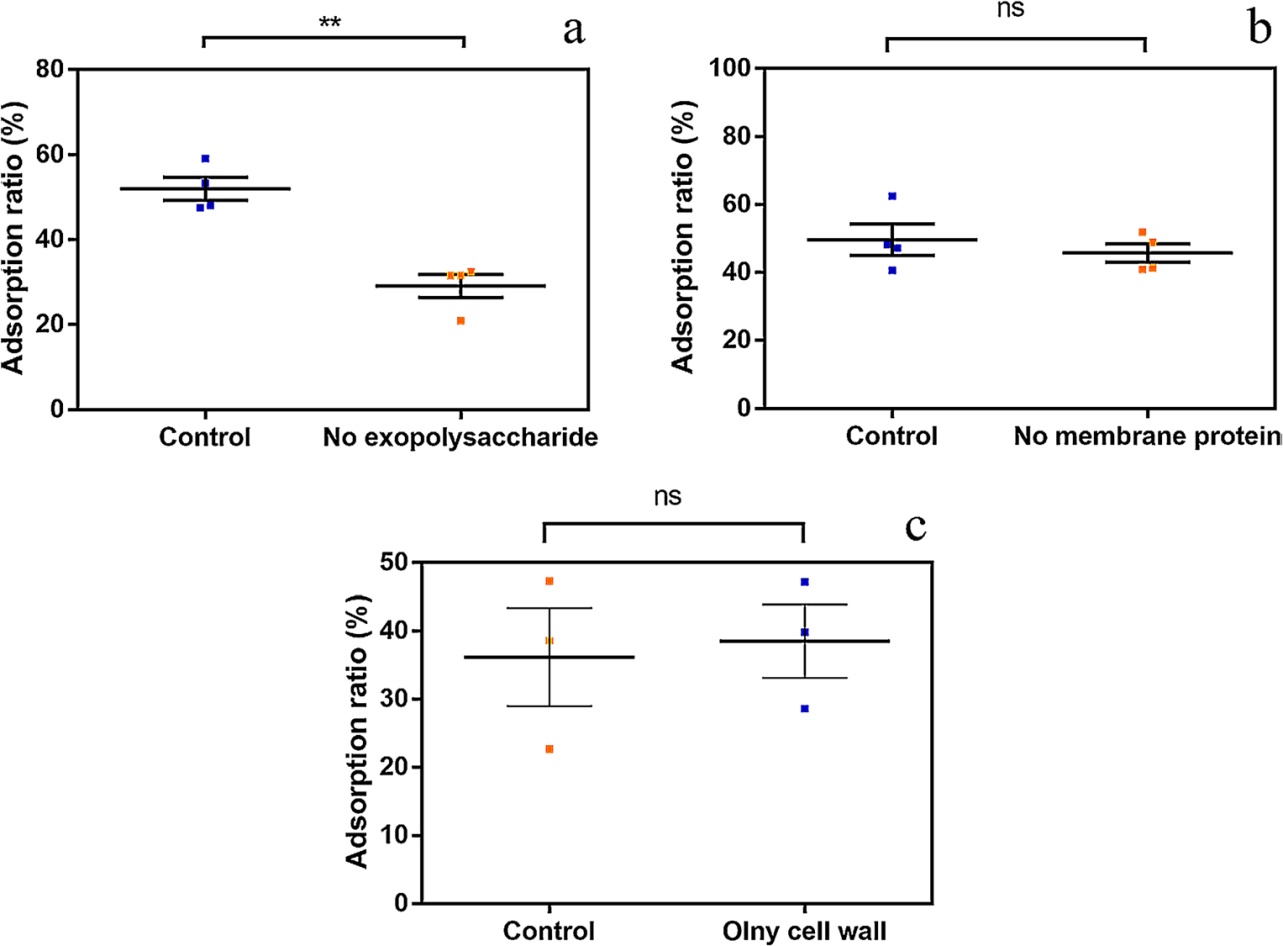
Adsorption ratios of DBP in different treatments in *L. plantarum* CGMCC18980. (**a**) Before and after exopolysaccharide removal (“**” means *P*<0.01). **b** Before and after membrane proteins removal. (**c**) Cell wall and intact cell

The effect of different cellular components of *L. plantarum* CGMCC18980 on DBP adsorption ratio was displayed in Fig. 3. When exopolysaccharide was removed, a significant reduction to 30% in adsorption ratio was observed, almost half of the original ratio (Fig. 3a) and the adsorption ratio was significantly reduced (*P*<0.01). However, without membrane protein and only cell wall had little effect on adsorption compared with normal cells (Fig. 3b and c). Their adsorption ratio was around 50%; there was no significant difference (*P*>0.05).

### FTIR analysis

**Fig. 4:**
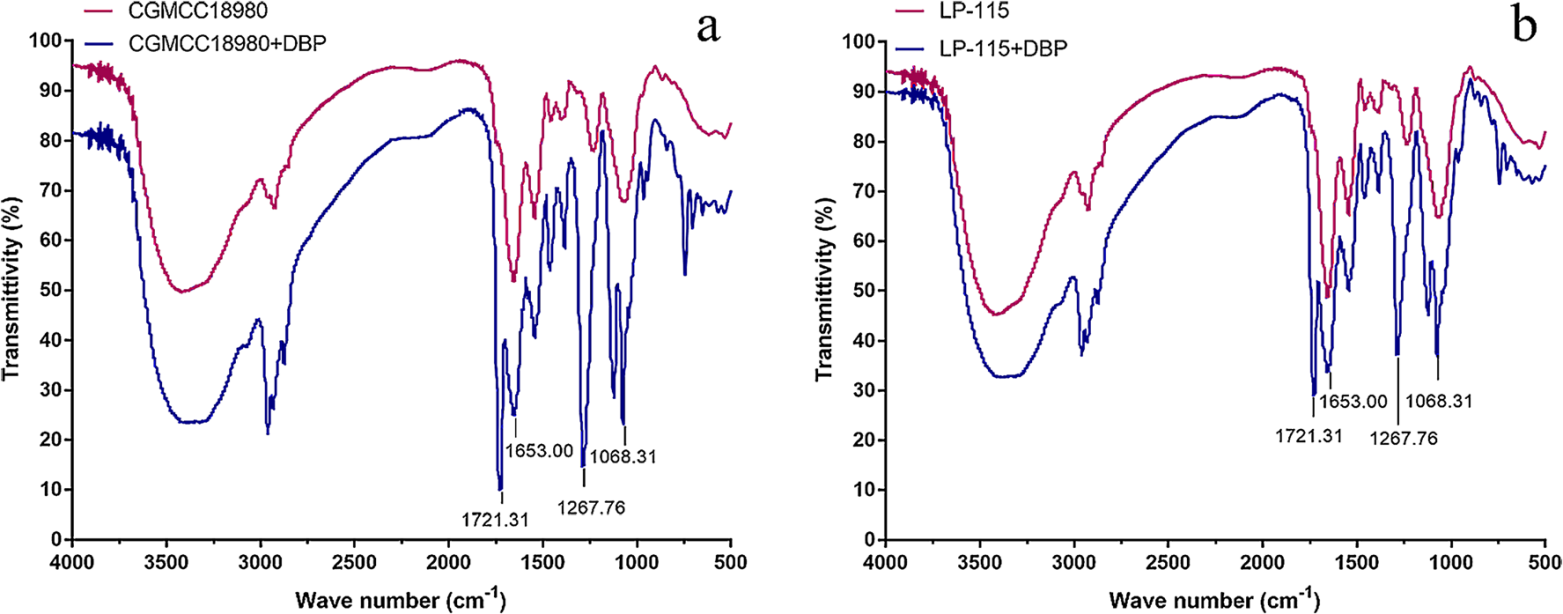
FTIR absorption spectra of two strains before and after DBP adsorption: **a** *L. plantarum* CGMCC18980; **b** *L. plantarum* LP-115

FTIR spectroscopy is a useful tool to identify the correlation between functional groups and adsorption capacity. The results of FTIR showed changes before and after adsorption in two strains (Fig. 4). After adsorption, the surface transmittance of bacterial cells in *L. plantarum* CGMCC18980 dropped from 88.70% to 14.70% at 1270 cm^−1^ (Fig. 4a). This phenomenon was similar to LP-115 (Fig. 4b). The results indicated that similar functional groups changed greatly after the adsorption in two strains. The assignments of FTIR bands and detailed wavenumber shifts for two strains are summarized in Table 1. The results displayed strong band at 1653.00 cm^−1^ and 1068.31 cm^−1^, and there was a possibility of overlap of the C=O, C-N, and C-O stretching vibrations. Those adsorption peaks were mainly from lipids and polysaccharides. Once adsorbed, the transmittance of CGMCC18980 was lower than that of LP-115, indicating that there was more DBP adsorbed on the cell wall of CGMCC18980.

**Table 1:** FTIR bands observed form DBP-exposed bacterial cells and DBP-unexposed bacterial cells

Functional groups	Wave number(cm^−1^)			
	CGMCC18980	CGMCC18980+DBP	LP-115	LP-115+DBP
O–H/N–H stretching	3428.96	3428.96	3428.96	3428.96
C–H stretching	2928.79	2928.79	2928.79	2928.79
C=O amide 1	1653.00	1653.00	1653.00	1653.00
N–H amide 2	1535.33	1535.33	1535.33	1535.33
O–H deformation	1461.96	1461.96	1461.96	1461.96
C–N stretching	1267.76	1267.76	1267.76	1267.76
C–O polysaccharides	1068.31	1068.31	1068.31	1068.31
C–X Alkyl Halide	724.04	724.04	724.04	724.04

### Zeta potential analysis

Zeta potential results of changes in cell surface potential before and after treatment were demonstrated in Table 2. The experimental results were −22.86 mV and −21.13 mV in *L. plantarum* CGMCC18980 before and after adsorption, and LP-115 were higher slightly. The bacteria cells were negatively charged and the adsorption system was relatively stable. But there was no significant difference showed between two strains (*P*>0.05), which indicated that DBP adsorption had little relationship with cell wall surface charge to some extent.

**Table 2:** Zeta potential (mV) before and after DBP adsorption of cells with pH = 4.0

Strains	Zeta potential (mV)	
	Before DBP adsorption	After DBP adsorption
CGMCC18980	−22.86±0.59^a^	−21.13±0.31^a^
LP-115	−20.20±0.26^a^	−20.90±0.44^a^

^a^The same letter means that there is no significant difference between the groups (*P* > 0.05)

### Characteristics of bacterial cells

**Fig. 5:**
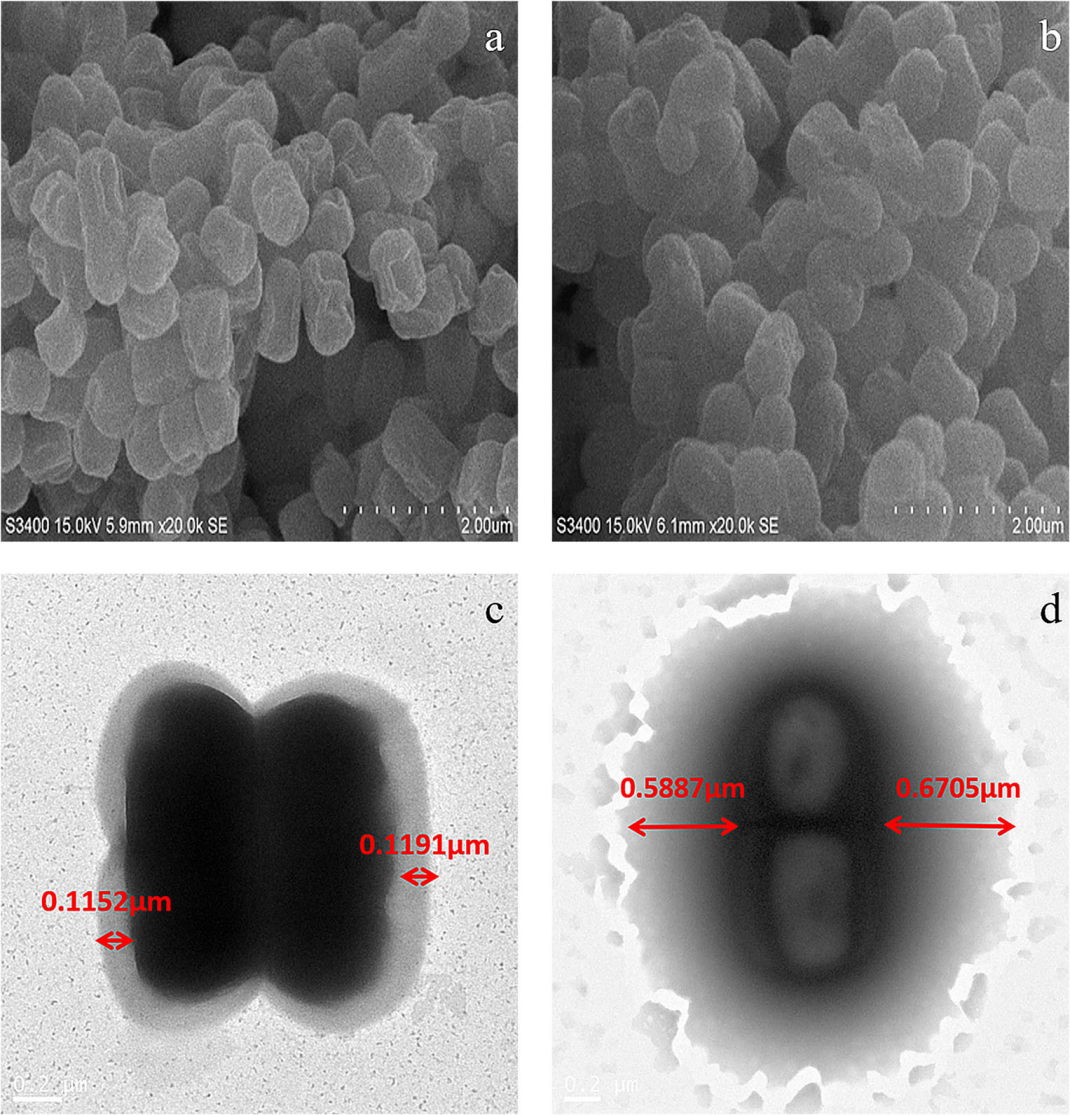
Electron microscopy images of *L. plantarum* CGMCC18980: (**a**) SEM images; (**b**) SEM images after adsorption; (**c**) TEM images; (**d**) TEM images after adsorption

Scanning electron microscopy (SEM) and transmission electron microscopy (TEM) were used to investigate the surface morphology of *Lactobacillus plantarum* CGMCC 18980 before and after incubation with DBP (Fig. 5). The surface of cells adsorbing DBP became smoother than the untreated one after treatment (Fig. 5a and b). By comparing the electron microscope results of CGMCC18980 before and after (Fig. 5c and d), it was clearly observed that the adsorption layer was changed from 0.1172±0.0019 to 0.6296±0.0409 μm.

## Discussion

The experimental results expounded that the adsorption ratio of *L. plantarum* CGMCC18980 on DBP reached 58.63% at the optimal conditions. Compared with the best results of 45.00% in *Leuconostoc mesenteroides* DM1-2 (Zhao et al. 2017) and 24.34% in *Lactobacillus plantarum* CCFM436 (Tong et al. 2016), CGMCC18980 was considered to have a high adsorption ability. When the cell proliferation reached a certain level, the adsorption ratio basically did not change, which was consistent with other reports (Piotrowska 2014). The phenomenon that adsorption ratios decreased with the increase of DBP concentration was similar to *Lactobacillus acidophilus* in adsorbing aflatoxin (Di Gregorio et al. 2017). In the previous 2.5 h, most of the DBP-binding were completed, which indicated that adsorption was a rapid process (Oluwafemi and Da-Silva 2009). The adsorption ratio fluctuated less based on the results considering temperature in this study. Many literatures focusing on aflatoxin (Haskard et al. 2001), bisphenol A (Endo et al. 2007), and zearalenone (Vega et al. 2017) have been reported. In this study, CGMCC18980 performed good adsorption capacity of DBP.

Through optimizing the adsorption conditions, measuring the changes of functional groups on the cell surface, and analyzing the components on the cell surface, exopolysaccharide was considered to play a key role in adsorption, and *L. plantarum* CGMCC18980 showed an excellent ability of adsorbing DBP. Electron microscopy results revealed the adsorption of DBP intuitively. FTIR data explained that the C=O, C-N, and C-O groups of two strains varied a lot, and it was related to lipids and polysaccharides. These results agreed with other reports about the adsorption of toxin and patulin (Guo et al. 2013; Hatab et al. 2012). However, some people believed that amino and carboxyl groups of bacterial cell walls were the main reason for the bacteria to bind to mycotoxin (Hatab et al. 2012). Others also thought that both protein and polysaccharides components on cell walls were involved in toxin removal (Alaleh et al. 2014; Wang et al. 2015). Although the zeta potential data showed no significant difference between two strains, the slight difference of surface charge may be caused by the cell wall structure (Martinez et al. 2008). Similar results have been found by other researchers when they studied the adsorption mechanism of patulin (Guo et al. 2013) and of aflatoxin B_1_ (AFB_1_) removal by adsorption in LAB (Haskard et al. 2000).

**Fig. 6:**
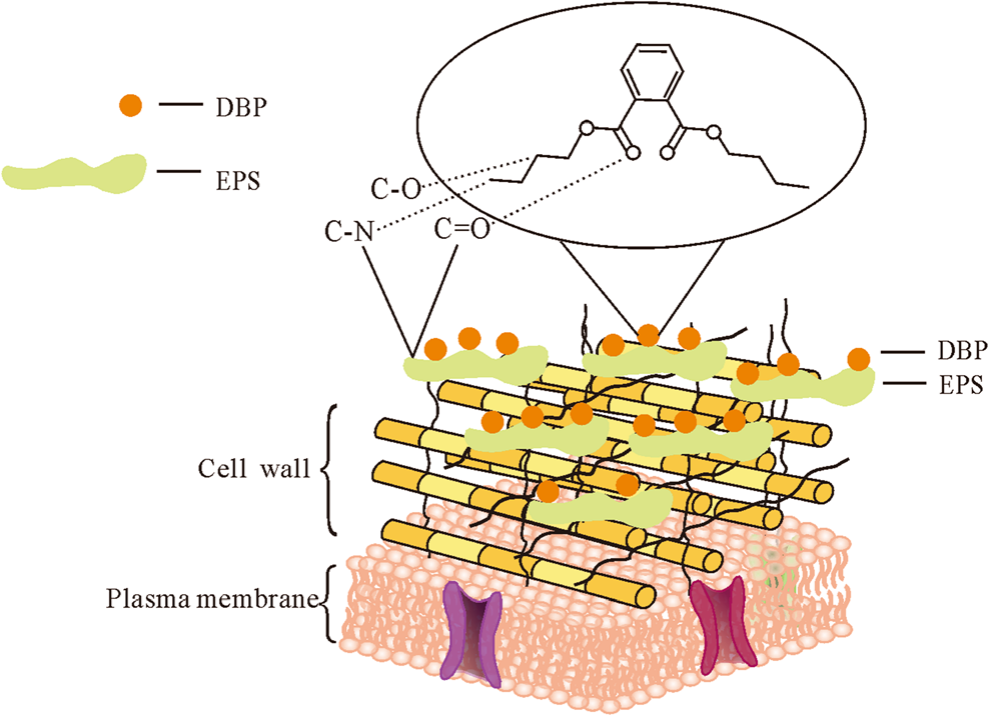
Overview of the potential DBP-adsorption mechanism

The high affinity occurred in *L. plantarum* CGMCC18980 may attribute to more adsorption sites in itself, especially in exopolysaccharide. An overview of probable adsorption mechanism in CGMCC18980 during the process was illustrated in Fig. 6. According to the results mentioned above, most of DBP was captured by exopolysaccharide on cell wall, relating to the functional groups of C-O, C-N, and C=O. It was speculated that exopolysaccharide was the key portion involved in adsorption.

In this study, *L. plantarum* CGMCC18980 was testified to have observably adsorption ratio of DBP, and the exopolysaccharide was probably an important factor of adsorption. Harmful substances are ubiquitous, like aflatoxin M1(AFM_1_) contamination found in Iranian cheese and Portuguese yogurt (Kamkar 2006; Martins and Martins 2004), and subsequent research considered that LAB could effectively reduce the free AFM_1_ content in liquid medium and yogurt processing (El Khoury et al. 2011). Biosorption is regarded as a promising method of the treatment and LAB belongs to GRAS so that it can be prepared into probiotic powders or applied to specific functional foods as a potential biological remover. By ingesting these products, consumers can make LAB exhibit its ability to reduce hazardous substances and drop the damages to body’s endocrine system eventually. What’s more, if the cells applied to specific foods in the future, further consideration should be given to whether the LAB can still maintain effects after fermentation.

*L. plantarum* CGMCC18980, which screened by our laboratory, showed excellent ability in the adsorption of DBP. Results of FTIR suggested that the functional groups C=O, C-N, and C-O, which related to lipids or polysaccharides, were involved in DBP adsorption. Furthermore, our results revealed that exopolysaccharide played a key role in adsorption. Obvious differences could be seen before and after adsorption through electron microscope observation, and *L. plantarum* CGMCC18980 showed obvious adsorption layer, while the zeta potential results exposed that DBP adsorption had nothing to do with the bacterial surface charge. Admittedly, the adsorption process of DBP is quite complex, and our investigation is only a small part and not comprehensive enough indeed. More deeper elements and factors need to be taken into account, such as the composition and structure of exopolysaccharide, as well as other components of the cell wall. In the further study, more detailed mechanism needs to be explained. Once the mechanism of action is revealed, it is considerable and promising to be applied to functional foods or potential drugs that are beneficial to health in the future.

## Data Availability

All data generated or analyzed during this study are included in this published article.
